# Insulin-Regulated Aminopeptidase in Women with Breast Cancer: A Role beyond the Regulation of Oxytocin and Vasopressin

**DOI:** 10.3390/cancers12113252

**Published:** 2020-11-04

**Authors:** María Jesús Ramírez-Expósito, Basilio Dueñas-Rodríguez, María Pilar Carrera-González, Joaquín Navarro-Cecilia, Jose Manuel Martínez-Martos

**Affiliations:** 1 Experimental and Clinical Physiopathology Research Group, Department of Health Sciences, School of Experimental and Health Sciences, University of Jaén, E-23071 Jaén, Spain; mramirez@ujaen.es (M.J.R.-E.); bdr@quesadasolidaria.org (B.D.-R.); pcarrera@uco.es (M.P.C.-G.); dr.jnavarro@hotmail.com (J.N.-C.); 2 Unit of Breast Pathology, Complejo Hospitalario de Jaén, E-23007 Jaén, Spain; 3 Department of Nursing, Pharmacology and Physiotherapy, Faculty of Medicine and Nursing, Instituto Maimónides de Investigación Biomédica de Córdoba, University of Cordoba, 14004 Córdoba, Spain

**Keywords:** angiotensin IV, AT4 receptor, oxytocin, BC, menopause, neoadjuvant

## Abstract

**Simple Summary:**

Insulin-regulated aminopeptidase (IRAP) is a well-known enzyme involved mainly in the regulation of the peptide hormones, oxytocin and vasopressin. However, this enzyme activity has hardly been analyzed in breast cancer patients. Additionally, the influence of both the hormonal status (pre or postmenopause) and the administration of neoadjuvant chemotherapy have rarely been studied. We show that there is a weak association between IRAP activity and the circulating levels of peptide hormones with variations depending on the hormonal status and the neoadjuvant treatment, and propose a role beyond oxytocin and vasopressin regulation that is related to the local mammary renin-angiotensin system and glucose transportation to the cells.

**Abstract:**

Insulin-regulated aminopeptidase (IRAP) is the only enzyme known to cleave oxytocin and vasopressin; however, it is also the high-affinity binding site for angiotensin IV (AngIV) receptor type 4 (AT4) ligands and it is related to insulin-dependent glucose transporters through the translocation of the glucose transporter type 4 (GLUT4). Previous studies have demonstrated an association between IRAP activity and the number and size of mammary tumors in an animal model of breast cancer (BC). Also, a highly significant increase in IRAP activity has been found in BC tissue from women patients. Here, we found no changes in circulating IRAP in premenopausal (preMP) women, but it increased significantly in postmenopausal (postMP) women not treated with neoadjuvant chemotherapy (NACH). However, in women treated with NACH, IRAP activity increased in both preMP and postMP women. Two years of follow-up indicated lower levels of IRAP activity in untreated preMP women, but a return to control levels in untreated postMP women, while IRAP activity returned to control levels in women treated with NACH. Circulating oxytocin decreased in both preMP and postMP women during the follow-up period. Differences in Oxytocin appeared between preMP and postMP women treated with NACH, but not in women who were not treated with NACH. On the contrary, circulating vasopressin increased in untreated and treated preMP and postMP women, with most of the differences related to the hormonal status as well as the neoadjuvant treatment during the two year follow-up We propose that IRAP is involved in mechanisms related not only to oxytocin and/or vasopressin regulation, but also to the local mammary RAS through AngIV and its role in glucose transportation through the IRAP/GLUT4 system.

## 1. Introduction

Aminopeptidases are enzymes involved in the catabolism of peptidic hormones, which have important physiological functions and act through autocrine and/or paracrine means. A broad range of peptide substrates have been described for most of them, thus suggesting a low functional specificity. However, we have clearly demonstrated that modifications in aminopeptidase-specific activities reflect the specific, local functional status of their corresponding substrates under different physiological and pathological conditions, both in vitro and in vivo and in animal models and humans. Several aminopeptidases have been found to be involved in a number of breast cancer (BC) cell lines, in the animal model induced by nitrosourea and in human patients [1,2,3,4,5,6]. These enzymes can be selectively modified in the affected tissue as a result of the specific microenvironment promoted by the tumoral cells.

Insulin-regulated aminopeptidase (IRAP), also called oxytocinase, cystyl aminopeptidase or placental leucyl aminopeptidase, is a membrane-bound zinc-metallopeptidase with many important regulatory functions. IRAP cleaves several peptide hormones in vitro including vasopressin, oxytocin, lys-bradykinin, angiotensin III (AngIII) and angiotensin IV (AngIV), and the neuropeptides met-enkephalin and dynorphin A [7,8,9,10]. However, IRAP is the only membrane aminopeptidase known to cleave vasopressin and oxytocin hormones in vivo [9,10,11,12,13]. Although IRAP was first described in human placenta, immunohistochemical analyses have shown it is present in a variety of tissues such as vascular endothelium, gastrointestinal mucosa, pancreas, bile duct, bronchial epithelium, renal tubules, sweat glands, adipocytes, skeletal muscle [14] and mammary gland [2]. Therefore, IRAP is not only involved in pregnancy, but also in several other physiological processes. IRAP is also known to be involved in other biological functions such as the presentation of antigens, the regulation of oxytocin and vasopressin levels and the translocation of the glucose transporter type 4 (GLUT4) [9,15,16,17,18]. Thus, IRAP is found in GLUT4 vesicles in insulin-responsive cells [19], and both molecules are translocated from these vesicles to the cell surface in response to certain stimuli such as insulin [15,20]. In addition, the existence of GLUT4, including BC cells, has been shown in non-insulin-dependent tissues such as the mammary gland, and their role in cell cycle progression has also been demonstrated [21,22]. In addition, several authors [23,24,25] have reported that IRAP is the specific high-affinity binding site for AngIV receptor type 4 (AT4) ligands. Ascher et al. [26] demonstrated that IRAP has a second Zn^2+^ binding site not associated with the catalytic region, which is lost with AngIV binding, and which might hinder the catalytic function of IRAP on its substrates. Several putative mechanisms have been suggested to explain the role of IRAP in mediating the actions of AT4 receptor ligands; in particular, some studies have indicated that competitive inhibition of IRAP catalytic activity promotes the increased availability of AT4 receptor endogenous ligands [27,28].

In a previous work using an animal model of BC induced by N-methyl-nitrosourea (NMU), we demonstrated a relationship between circulating IRAP activity and the number and size of mammary tumors. We have also described a highly significant increase in this activity in tumoral tissue in women with BC [29]. In the present work, we analyzed circulating IRAP activity in premenopausal (preMP) and postmenopausal (postMP) women with BC treated or not with neoadjuvant chemotherapy (NACH) just before surgery and after two years of follow-up. We evaluated not only its role in the progression of BC, but also the putative value of this enzyme activity as a biological marker of the evolution of the disease and the response to treatment. In addition, we analyzed the circulating levels of oxytocin and vasopressin to understand the association between IRAP activity and its role as a regulator of oxytocin/vasopressin hormones in human BC. Oxytocin is normally found in cells of healthy mammary tissue but is rarely or never detected in breast tumors [30]. In fact, oxytocin inhibits the proliferation of human BC cell lines in vitro [31], in animal models [32,33,34] and in women [35,36], and thus it may play a role in the prevention of this disease. On the other hand, vasopressin has been reported to have a proliferative effect on cancer cells and it seems that the release of vasopressin by these cells is involved in tumor growth and survival among other steps of cell proliferation [37,38,39,40,41].

## 2. Results

### 2.1. Circulating Specific IRAP Activity

**Figure 1:**
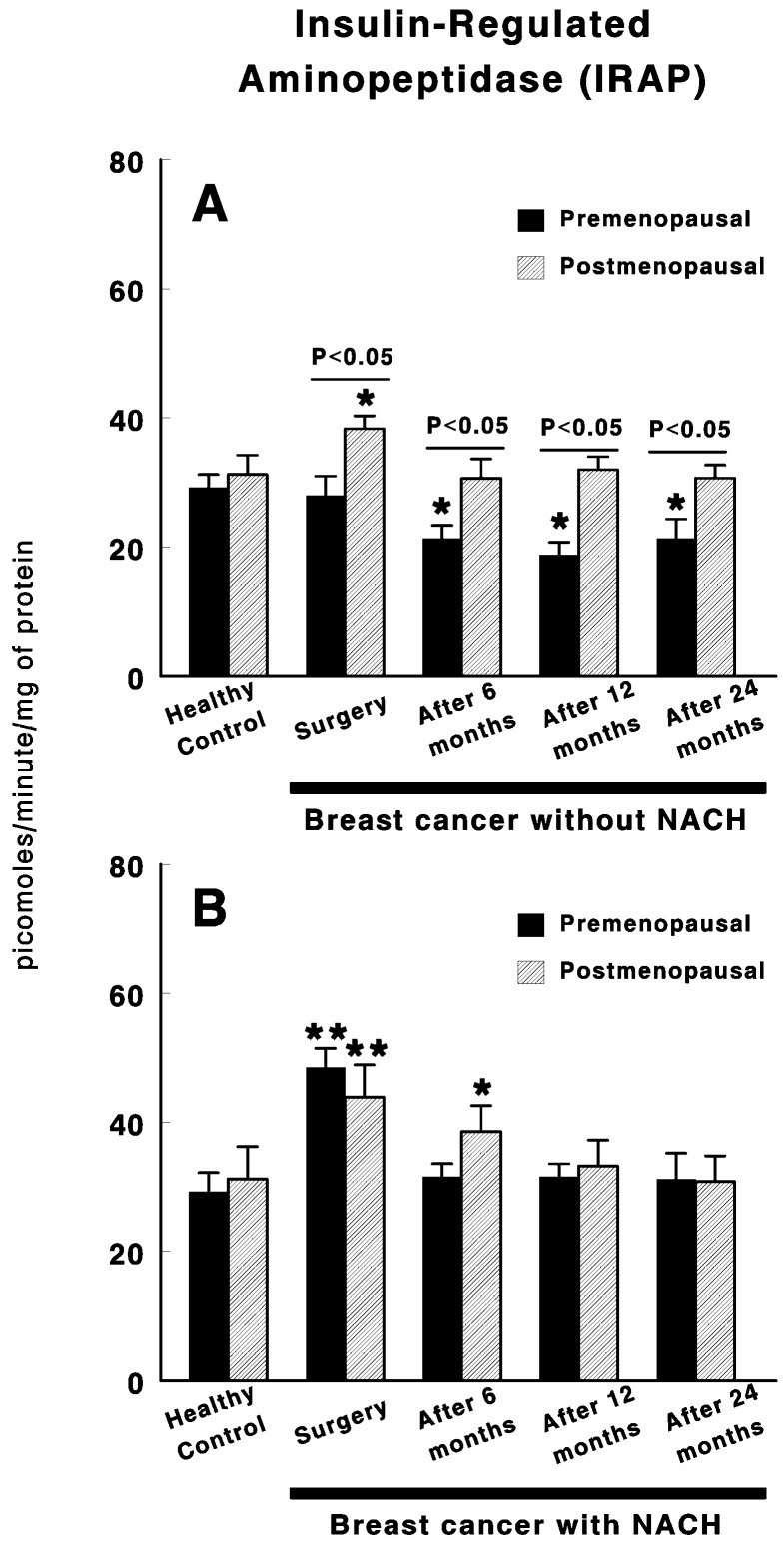
Specific serum insulin-regulated aminopeptidase (IRAP) activity in control, premenopausal (preMP) and postmenopausal (postMP) women and in women diagnosed with ductal infiltrating breast cancer (BC) who were untreated (**A**) or treated (**B**) with neoadjuvant chemotherapy (NACH), at the time of the surgery and at 6, 12 and 24 months after surgery. Asterisks represent significant differences between breast cancer patients and healthy control women. The upper lines between bars represent the significant differences between premenopausal and postmenopausal women. Results are expressed in picomoles of leucyl-β-naphthylamide hydrolyzed per min and per mg of protein (Mean ± SEM; * *p <* 0.05; ** *p <* 0.01).

Figure 1 shows the results obtained for serum IRAP specific activity in control preMP and postMP women and in women with ductal infiltrating BC treated or not with NACH, at the time of the surgery and 6, 12 and 24 months after surgery. In women not treated with NACH, no changes were found in IRAP specific activity in preMP women at the time of the surgery. However, a significant decrease in specific IRAP activity (*p <* 0.05) was found 6 months after surgery. These values remained significantly low (*p <* 0.05) up to 24 months after surgery (Figure 1A). Significant differences remained between preMP and postMP women. On the contrary, a significant increase (*p <* 0.01) was found in IRAP specific activity levels in postMP women with BC at the time of surgery, which returned to control levels 6 months after surgery and did not change up to 24 months after surgery (Figure 1A). On the contrary, in women with BC treated with NACH, IRAP activity significantly increased (*p <* 0.01) in both pre and postmenopausal women at the time of the surgery (Figure 1B). These values returned to control levels 6 months after surgery in preMP women and 12 months after surgery in postMP women. In both groups of women, the values remained unchanged up to 24 months after surgery (Figure 1B).

### 2.2. Circulating Oxytocin

**Figure 2:**
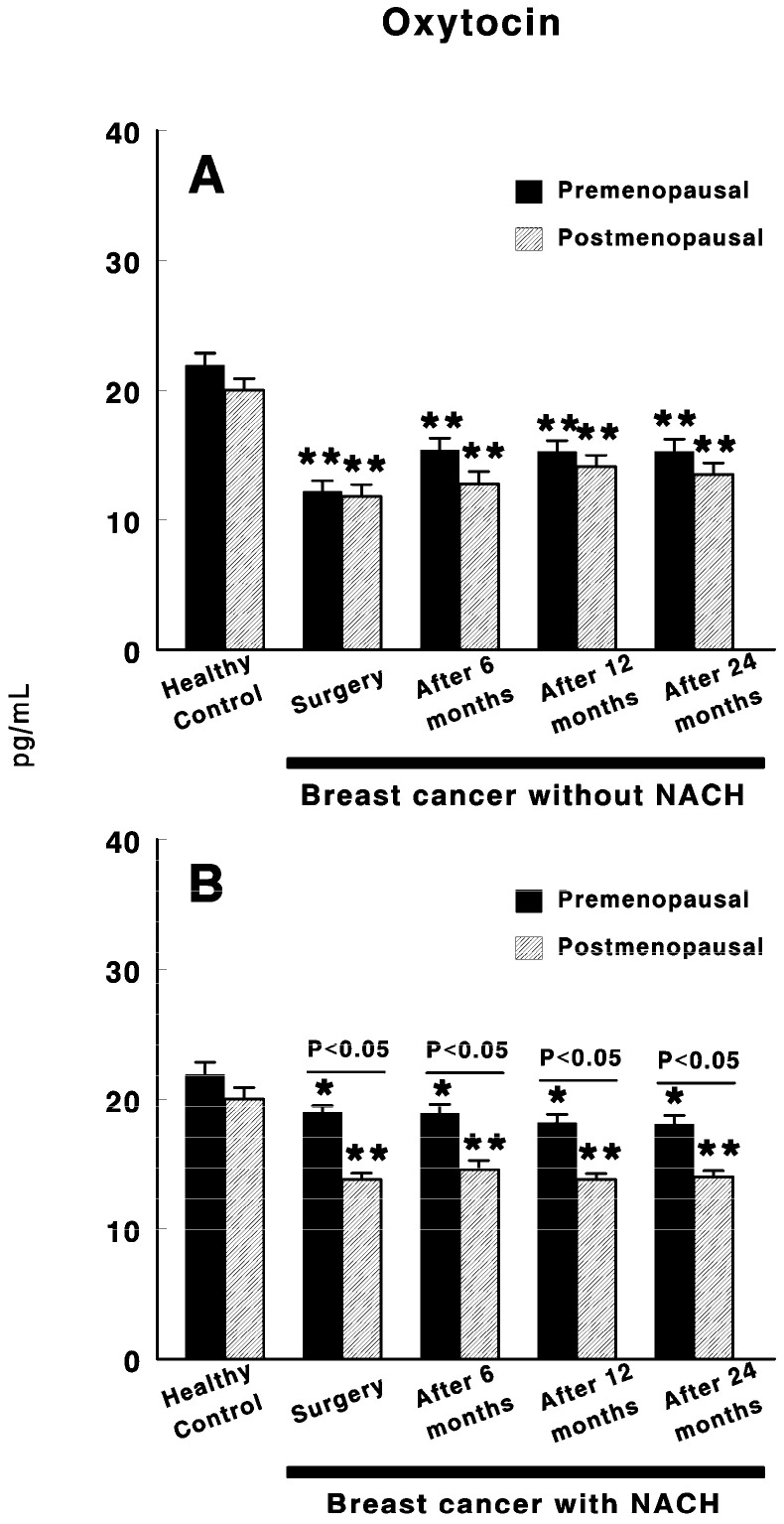
Circulating oxytocin levels in control, preMP and postMP women and in women diagnosed with ductal infiltrating BC who were untreated (**A**) or treated (**B**) with NACH, at the time of the surgery and at 6, 12 and 24 months after surgery. Asterisks represent significant differences between breast cancer patients and healthy control women. The upper lines between bars represent the significant differences between premenopausal and postmenopausal women. Results are expressed in pg/mL (Mean ± SEM; * *p <* 0.05; ** *p <* 0.01).

Figure 2 shows oxytocin levels in control preMP and postMP women and in women with BC treated or not with NACH, at the time of the surgery and 6, 12 and 24 months after surgery. In women not treated with NACH, a significant decrease (*p <* 0.01) in oxytocin levels was found in both pre and postmenopausal women at the time of the surgery when compared with control women. These values remained low in both groups of women up to 24 months after surgery. However, slightly significant higher levels were found in preMP–but not postMP women 6 to 24 months after surgery when compared with the values obtained at the time of the surgery (Figure 2A).Additionally, in women with BC treated with NACH, there was a significant decrease (*p <* 0.01) in oxytocin levels in both pre and postmenopausal women at the time of the surgery when compared with control women (Figure 2B). These values remained low in both groups of women up to 24 months after surgery. However, oxytocin levels are significantly (*p <* 0.05) lower in postMP women than in preMP at the time of the surgery and up to 24 months after surgery, although no significant differences were found in control preMP vs. postMP women (Figure 2B).

### 2.3. Circulating Vasopressin

**Figure 3:**
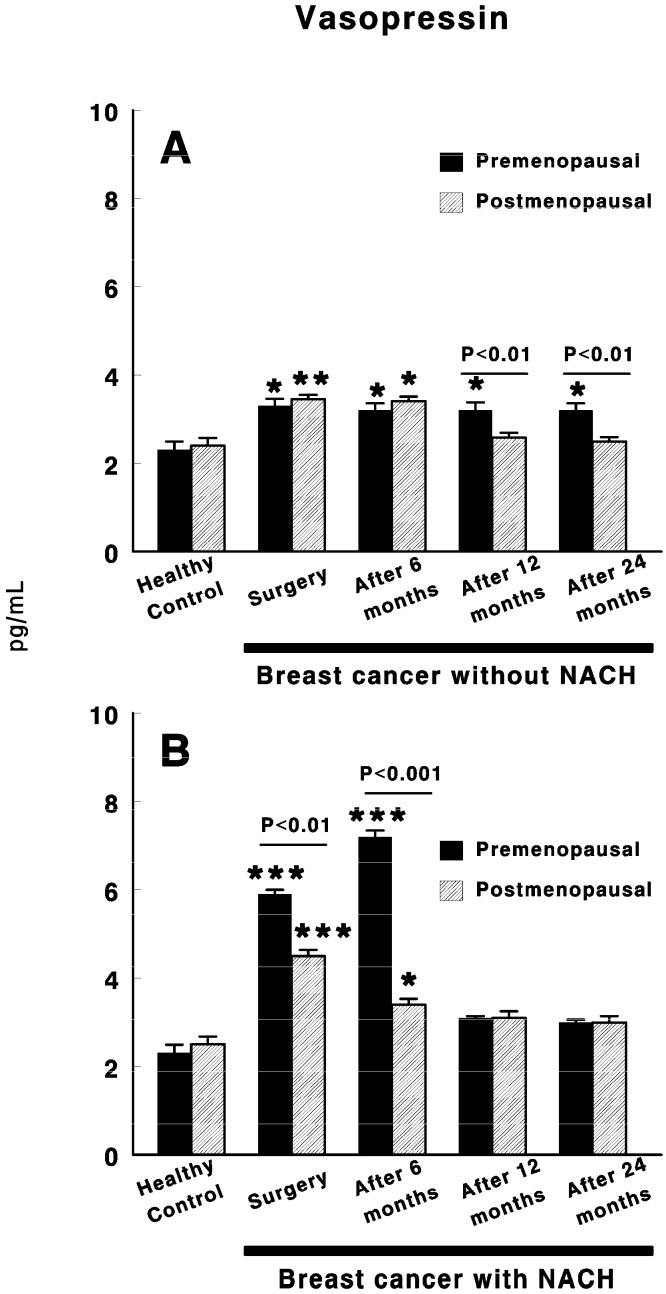
Circulating vasopressin levels in control, preMP and postMP women and in women diagnosed with ductal infiltrating BC that were untreated (**A**) or treated (**B**) with NACH, at the moment of the surgery and 6, 12 and 24 months after surgery. Asterisks represent significant differences between breast cancer patients and healthy control women. The upper lines between bars represent the significant differences between premenopausal and postmenopausal women. Results are expressed in pg/mL (Mean ± SEM; * *p <* 0.05; ** *p <* 0.01; *** *p <* 0.001).

Figure 3 shows the vasopressin levels in control preMP and postMP women and in women with ductal infiltrating BC who were treated or not with NACH, at the time of the surgery and 6, 12 and 24 months after surgery. In women not treated with NACH, there was a significant increase in vasopressin levels in both preMP (*p <* 0.05) and postMP (*p <* 0.01) women at the time of the surgery when compared with control women. These values remained high in both groups of women up to 6 months after surgery in postMP women, but up to 24 months after surgery in preMP women (Figure 3A), with significant differences appearing (*p <* 0.01) between the preMP and postMP women. Similarly, in women with BC treated with NACH, a significant increase (*p <* 0.001) in vasopressin levels was found in both pre and postmenopausal women at the time of the surgery when compared with control women, with the values in preMP women being significantly higher (*p <* 0.01) than in postMP women. These levels remained significantly high in both groups of women (*p <* 0.001 in preMP women and *p <* 0.05 in postMP women) with significant differences between them (*p <* 0.001) up to 6 months after surgery. However, vasopressin levels returned to control values in both preMP and postMP women 12 months after surgery and this remained constant up until the end of the study at 24 months (Figure 3B).

## 3. Discussion

The present work shows significant changes in circulating IRAP specific activity in women with BC to different degrees and in different directions depending on the hormonal status (pre or postmenopausal women) and the administration or not of NACH. IRAP could have an important role in BC in relation to its participation in the local renin-angiotensin system (RAS) at the mammary level. Albiston and coworkers identified the IRAP enzyme as the angiotensin IV (AngIV) type four receptor (AT4) [23,24]. The local RAS has been linked to several of the hallmarks of cancer, which are related to the process whereby normal cells become cancer cells. This includes all of the multiple steps that allow tumoral cells to survive, proliferate and disseminate. In fact, the most well-known RAS signaling is mediated by angiotensin II (AngII) through its type 1 (AT_1_) receptor, and increases cell proliferation and modulates the growth of vascular cells during angiogenesis [42]. However, little or no information is available about the other angiotensin peptides. Previous studies by our group have described changes in the metabolism of angiotensin in preMP and postMP women with BC that suggested the formation of AngII, although this was through different pathways involving different aminopeptidases depending on the hormonal status [5,6]. AngII production and/or expression is associated with improvements in BC-related adverse outcomes [43,44]. On the contrary, treatment with NACH promotes AngII catabolism independently of the hormonal status, which minimizes its role as a tumor promotor and even inhibits tumor growth. Our results also suggest an increase in circulating levels of AngIV, and therefore, of the IRAP substrate. Here we show that changes in specific IRAP activity occurs with BC in untreated postMP women, and in pre and postmenopausal women treated with NACH, including changes that are evident after a follow-up of two years. In addition, it must be taken into account that mammalian cells use glucose as their main source of energy production. The presence of GLUT4 has been demonstrated in tissues that are independent of insulin, such as the mammary gland, and also in BC cells. In fact, its role in cell cycle progression has been shown [21,22]. Indeed, Garrido et al., [45,46] confirmed the importance of GLUT4 in the estrogen-dependent metabolism of MCF-7 human BC cells. Thus, the inhibition of the GLUT4 inhibits cell proliferation and decreases cell viability when hypoxic conditions occurs. This provides a proof-of-principle for the feasibility of using pharmacological approaches to inhibit GLUT4 in order to induce metabolic reprogramming in vivo in BC models [46]. As mentioned previously, both GLUT4 and IRAP are located in specialized vesicles, which require a stimulus to translocate to the cell membrane. According to Garrido’s studies, the differences observed by us in the specific IRAP activity will determine its bioavailability depending on the hormonal status of the patients, the tumoral process and other stimuli that could be involved in the translocation and/or availability of the enzyme throughout the two years studied. Our previous findings in women with BC also suggested an increase in circulating levels of AngIV, whose actions are mediated by the AT4 receptor [20], and AT4 receptor ligands dose-dependently inhibit the catalytic activity of IRAP [23,24].

**Figure 4:**
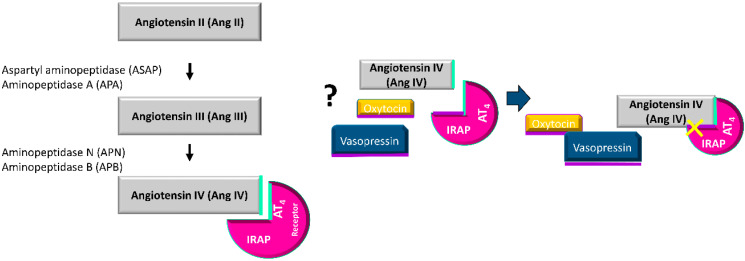
Potential regulation of the catalytic activity of IRAP by the binding of angiotensin IV (AngIV).

Here, we also determined the levels of the best-known IRAP substrates: oxytocin and vasopressin. Circulating levels of oxytocin and vasopressin differentially change depending on the hormonal status and the administration of neoadjuvant therapy. However, a weak association exists between circulating IRAP activity and circulating oxytocin or vasopressin levels, but this association occurs to a higher degree with vasopressin compared to oxytocin. It must be taken into account that IRAP is the only enzyme known to cleave vasopressin [11,13,47]. Thus, the regulatory functions of IRAP activity may not be exclusively related to, but may go beyond oxytocin and/or vasopressin regulation. Gupta and coworkers [48] studied serum IRAP in patients with breast tumors and found an increase in the enzyme with clinical staging. Furthermore, enzyme activity was correlated with the histological subtypes of breast carcinoma. In fact, these authors proposed IRAP as a sensitive prognostic indicator of invasiveness in breast carcinoma. We have also previously described significant changes in circulating IRAP activity in rats with mammary tumors induced by NMU [1,49]. The levels of activity in this model correlated with the number and size of the tumors and also indicated a lower availability of oxytocin. Low oxytocin levels from both animal models and women, as shown in the present work, could explain the increase in the OTR number described by several authors in BC and the progression of the disease. In fact, our results show low levels of oxytocin in both preMP and postMP women at the time of surgery and up to two years after surgery, with these values being even lower in postMP women treated with neoadjuvant chemotherapy. Furthermore, these values remain unchanged despite surgery or the NACH treatment. We must also bear in mind that oxytocin, as has been described, inhibits tumor growth. Therefore, this statement would be in line with our results. Oxytocin plays multiple roles in mammals. It can act as a neurotransmitter, neuromodulator and hormone. The diverse roles of oxytocin are related to several signaling pathways. All of them are activated by its binding to its cell surface OTR, of which there is only one isoform [31,50]. Several authors have described the suppressive effect of oxytocin on breast tumor formation and development [32,33]. One study demonstrated that the administration of oxytocin reduces tumor growth through the reduction in the expression of proteins of the signaling pathways involved in cell proliferation and induction, such as Akt and ERK, and also through the inhibition of estrogen receptor (ER) expression [35]. It must be taken into account that nearly 75–80% of breast tumors are ER-positive tumors [51]. In an animal model of BC, the volume and weight of transplanted tumors were significantly decreased after oxytocin administration [34]. The observed decrease in oxytocin levels must be related to changes in OTR expression, which is contrary to what occurs for other type of hormone receptors whereby biological responses are modulated by changing the concentration of the hormones. Instead, the oxytocin/OTR system (Figure 4) is mainly regulated by changes in OTR expression [52,53], but OTR was not analyzed here. Therefore, the lack of adequate oxytocin levels could be responsible, at least in part, for the progression of the disease.

On other hand, it has been extensively reported that all BCs express the vasopressin gene, which leads to the release of both normal and aberrant forms of tumor vasopressin mRNA and proteins. In fact, Taylor et al. have suggested that since changing vasopressin concentrations can significantly influence the cell growth in MCF-7 cells, this peptide is likely to be an important modulator of growth in some human breast carcinomas [54]. Also, BC cells express normal genes for all vasopressin receptors. Through these receptors, vasopressin has a multifaceted effect on tumor growth and metabolism. Our data also support this fact, that is, there were increased levels of vasopressin in both pre and postmenopausal women at the time of surgery and up to 6–12 months after surgery in untreated women. In women treated with NACH, vasopressin levels were even higher up to 6 months after surgery in preMP women, but returned to control levels 12 months after surgery in both preMP and postMP women, which supports the idea of ending the stimulant growth of vasopressin in cancer cells.

It has been proposed that peptide production by tumors is part of a particular process of oncogenic transformation rather that a pre-existing condition in progenitor cells; this concept has been called the “Selective Tumor Gene Expression of Peptides Essential for Survival” (STEPS) [55]. The changes in IRAP specific activity found here together with the previous changes found in the NMU model of rats could also indicate the dysregulation of vasopressin functions. However, it should not be forgotten that this finding was observed in the absence of variations in plasma osmolality and/or in extracellular fluid levels, and thus it may be due to psychogenic stimuli such as fear or apprehension, which are well known to affect vasopressin release [56].

One important limitation of our study is that IRAP works as a membrane-bound enzyme that works in close proximity with its substrates/ligands. Here, we measured circulating IRAP specific activity, which may also reflect other additional effects of the tumoral process and/or treatments in our patients. In addition, we did not measure circulating AngIV levels and their association to IRAP activity. In any case, we can conclude that IRAP is involved in the mechanisms related not only to oxytocin and/or vasopressin regulation, but also to the local mammary RAS and its role in glucose transportation through the IRAP/GLUT4 system. Although, to our knowledge, our findings on the role of AngIV on BC have not been described previously in the literature, it has been reported that AngIV (and to a lesser extent, AngII) stimulates the activity of tyrosine kinases and, therefore, cell proliferation in estrogen-induced rat pituitary tumors [57].

## 4. Materials and Methods

### 4.1. Subjects and Study Design

A total of 198 women were recruited at the Unit of Breast Pathology at the University Hospital of Jaén, Spain and 78 women volunteers without carcinoma were also included as control groups. This study was approved by the Ethics Committee of the University Hospital of Jaén on 25 June 2018 (ethic code 0756-N-18) and every subject freely signed their consent. The clinicopathological characteristics (Table 1) of each patient include: age at diagnosis, tumor size, tumor histology, pathologic T classification, Scarff–Bloom–Richardson grade, hormonal and HER-2/neu status and molecular subtype. The immunohistochemical assessment was carried out at the time of diagnosis in every tumor block by a pathologist specialized in breast cancer. Paraffin embedded tumor specimens were analyzed using immunohistochemical methods to measure hormonal receptors (HRs), HER2 status and the Ki-67 labeling index. HER2 status was determined by the Dako HercepTest as well as by a fluorescence in situ hybridization test (FISH) in biopsies with a 2+ score via the immunohistochemical analysis. Tumors were classified as positive with a 3+ score via immunohistochemistry or a 2+ score via immunohistochemistry with a positive FISH analysis. Estrogen (ER) and progesterone (PR) receptors were measured using mononuclear antibodies (Master Diagnostica, clone 6F11, prediluted, and Master Diagnostica 1A6, prediluted, respectively). Tumors were classified as HR positive if ≥10% of the tumor cell nuclei stained positive. The Ki-67 labeling index was evaluated with a mononuclear antibody (Master Diagnostica, Granada, Spain; clone SP6, prediluted). Tumors were considered to have high rates of proliferation if ≥20% of the cell nuclei stained positive for Ki-67. The Ki-67 index was quantified by semiquantitative scale imaging as recommended by the American College of Pathologists. For results between 10% and 30%, 500 cells were used for better adjustment to the relevant cut-off percentage. The most peripheral tumor areas were selected as they correspond to the front infiltration of the neoplastic. Data on HR and HER2 expression were used to classify the histological subtypes of breast cancer. Depending on their tumor molecular type (luminal A, luminal B, Her2/neu or triple negative), patients received adjuvant therapies after surgery that include treatments with radiotherapy, immunotherapy (Her2/neu+) and hormone therapy (tamoxifen or aromatase inhibitors). As part of their routine checkups, blood samples were taken at 6, 12 and 24 months after surgery.

The women with BC were diagnosed with ductal infiltrating carcinoma. Of these women, 83 (39 preMP and 44 postMP) did not receive NACH, and 115 of them (63 preMP and 52 postMP) received NACH before surgery. The treatment of patients who received NACH was based on an anthracycline/taxane regimen which included four courses of EC (epirubicin 90 mg/m^2^ and cyclophosphamide 600 mg/m^2^, every twenty-one days), followed by eight courses of 100 mg/m^2^ paclitaxel once a week or four courses of 75 mg/m^2^ docetaxel every twenty-one days. Those patients with an overexpressing HER2/neu tumor also received trastuzumab (fourteen courses of 6 mg/kg every twenty-one days). Those women with triple-negative BC received six cycles of 75 mg/m^2^ docetaxel plus carboplatin (area under a curve of six).

Seventy-eight women, aged 28 to 69 years old were recruited as the control group including 38preMP women with regular menstrual periods, and 40 postMP women with spontaneous menopause for at least one year. None of the control women had a previous history of any type of cancer, chemotherapy, hormonal or antioxidant therapy, or chronic diseases. Their routine gynecological examinations, including mammography, were normal in all cases.

For both the control and patient groups, women were excluded if they were current smokers, regular alcohol consumers, used antioxidant supplements, pregnant or lactating, presented hepatic, cardiac or renal dysfunction, undergoing hormonal therapy, used drugs, or had hypertension, diabetes or other potentially chronic conditions.

### 4.2. Sample Acquisition

Samples of blood were obtained after an overnight fast by venous arm puncture in tubes without anticoagulants. They were allowed to clot and were centrifuged at 3000× *g*, for 10 min, at 4 °C to obtain the serum. Serum samples were collected, frozen in liquid nitrogen and kept at −80 °C until use.

### 4.3. Insulin-Regulated Aminopeptidase Activity Assay

Serum IRAP specific activity was measured fluorometrically using leucyl-β-naphthylamide (LeuNNap) as substrate, as previously described in [1]. Briefly, 10 µL of the sample (in triplicate) was incubated (30 min at 37 °C) with 100 µL of a substrate solution composed of 100 µM LeuNNap and 0.65 mM dithiothreitol (DTT) in 50 mM phosphate buffer, pH 7.4. The reaction was stopped by adding 100 µL of 0.1 M acetate buffer, pH 4.2. The β-naphthylamine released as the result of the enzyme activity was measured fluorometrically (λex: 345 nm; λem: 412 nm). Total protein was quantified in triplicate by using the Bradford method.

### 4.4. Oxytocin Assay

Samples were measured by an oxytocin ELISA (Assay Designs), according to the manufacturer’s instructions. The lower limit of assay detection is 0.09 ng/mL, the intra-assay coefficient of variation is between 2.6–3.3%, and the inter-assay coefficient of variation is between 6.1–7.3%.

### 4.5. Arginine-Vasopressin Assay

Samples were measured by an arginine-vasopressin ELISA (Enzo Life Sciences, Farmingdale, NY, USA), according to the manufacturer’s instructions. The lower limit of assay detection is 2.84 pg/mL, the intra-assay coefficient of variation is between 6.0–14.3% and the inter-assay coefficient of variation is between 6.4–9.5%.

### 4.6. Statistical Analysis

Differences between groups were analyzed by multiple analysis of variance plus the Newman–Keul post-hoc test, using IBM SPSS V.23 software (IBM Corp., Armonk, NY, USA). All comparisons with *p*-values below 0.05 were considered significant.

## 5. Conclusions

Circulating IRAP specific activity may be involved in the promotion and progression of BC in both preMP and postMP women through the regulation of oxytocin and vasopressin, although there is a weak association between them. In addition, NACH influences this enzyme activity and promotes the return of vasopressin, but not oxytocin, to control levels. Therefore, additional functions must be carried out by IRAP enzyme, and it probably acts as a regulator of the local mammary RAS cascade through AngIV and/or energy supply regulation.

## Figures and Tables

**Table 1:** Clinicopathological description of the patients involved in this study.

Characteristics	Premenopausal		Postmenopausal	
	Untreated	Treated with Neoadjuvance	Untreated	Treated with Neoadjuvance
	n (%)	n (%)	n (%)	n (%)
Age (Years)				
Mean	45.2 ± 1.2	45.1 ± 0.8	65.3 ± 0.9	65.3 ± 0.90
Median	48	46	64	63
Range	27–54	27–54	57–78	56–78
Tumor Histology				
Ductal	39 (100%)	63 (100%)	44 (100%)	52 (100%)
Lobular	0 (0%)	0 (0%)	0 (0%)	0 (0%)
Other	0 (0%)	0 (0%)	0 (0%)	0 (0%)
Molecular Subtypes				
Luminal A	23 (59.0%)	34 (54.0%)	27 (61.4%)	27 (51.9%)
Luminal B	10 (25.6%)	7 (11.1%)	6 (13.6%)	12 (23.1%)
Her-2	2 (5.1%)	18 (28.6%)	4 (9.1%)	0 (0%)
Triple negative	4 (10.3%)	4 (6.3%)	7 (15.9%)	13 (25.0%)
Pathologic Tumor Size (cm)				
Mean ± SEM	1.31 ± 0.09	3.02 ± 0.17	1.52 ± 0.14	3.36 ± 0.15
Median	1.20	3.00	1.30	3.00
Range	0.5–3.1	0.8–5.6	0.8–5.0	1.4–5.0
Pathologic T Classification				
0	0 (0%)	0 (0%)	0 (0%)	0 (0%)
1	35 (89.7%)	18 (28.6%)	40 (90.9%)	6 (11.5%)
2	4 (10.3%)	40 (63.5%)	4 (9.1%)	43 (82.7%)
3	0 (0%)	5 (7.9%)	0 (0%)	3 (5.8%)
Scarf–Bloom–Richardson Grade				
I	19 (48.7%)	8 (12.7%)	10 (22.7%)	13 (25%)
II	20 (51.3%)	55 (87.3%)	34 (77.3%)	39 (75%)
III	0 (0%)	0 (0%)	0 (0%)	0 (0%)
Hormonal Status				
ER+	33 (84.6%)	41 (65.1%)	33 (75.0%)	36 (69.2%)
ER−	6 (15.4%)	22 (34.9%)	11 (25.0%)	16 (30.8%)
PgR+	25 (64.1%)	41 (65.1%)	27 (61.4%)	33 (63.5%)
PgR−	14 (35.9%)	22 (34.9%)	17 (38.6%)	19 (36.5%)
HER-2/neu Status				
Negative	29 (74.4%)	38 (60.3%)	34 (77.3%)	49 (94.2%)
Positive	10 (25.6%)	25 (39.7%)	10 (22.7%)	3 (5.8%)
